# The Emerging Role of uORF-Encoded uPeptides and HLA uLigands in Cellular and Tumor Biology

**DOI:** 10.3390/cancers14246031

**Published:** 2022-12-07

**Authors:** Lara Jürgens, Klaus Wethmar

**Affiliations:** University Hospital Münster, Department of Medicine A, Hematology, Oncology, Hemostaseology and Pneumology, 48149 Münster, Germany

**Keywords:** non-canonical peptides, translation, uPeptides, uORFs, HLA uLigands, cancer, immunotherapy

## Abstract

**Simple Summary:**

The biological relevance of peptides that originate from non-canonical translational initiation sites have been increasingly recognized over the years. Peptides encoded by open reading frames upstream of canonical protein coding sequences are frequently translated and act as translational regulators, contribute to the immunopeptidome as cellular antigens, and are implicated in various cellular functions through peptide–protein interactions or as part of protein complexes. In this review, we first give an overview of the most relevant technical advances in non-canonical peptide detection. In the second part of the review, we focus on the functional implications of uPeptides and delineate how this largely unexplored compartment of the human peptidome affects tumor biology and may offer new opportunities for targeted and immunological cancer therapy.

**Abstract:**

Recent technological advances have facilitated the detection of numerous non-canonical human peptides derived from regulatory regions of mRNAs, long non-coding RNAs, and other cryptic transcripts. In this review, we first give an overview of the classification of these novel peptides and summarize recent improvements in their annotation and detection by ribosome profiling, mass spectrometry, and individual experimental analysis. A large fraction of the novel peptides originates from translation at upstream open reading frames (uORFs) that are located within the transcript leader sequence of regular mRNA. In humans, uORF-encoded peptides (uPeptides) have been detected in both healthy and malignantly transformed cells and emerge as important regulators in cellular and immunological pathways. In the second part of the review, we focus on various functional implications of uPeptides. As uPeptides frequently act at the transition of translational regulation and individual peptide function, we describe the mechanistic modes of translational regulation through ribosome stalling, the involvement in cellular programs through protein interaction and complex formation, and their role within the human leukocyte antigen (HLA)-associated immunopeptidome as HLA uLigands. We delineate how malignant transformation may lead to the formation of novel uORFs, uPeptides, or HLA uLigands and explain their potential implication in tumor biology. Ultimately, we speculate on a potential use of uPeptides as peptide drugs and discuss how uPeptides and HLA uLigands may facilitate translational inhibition of oncogenic protein messages and immunotherapeutic approaches in cancer therapy.

## 1. Introduction

The classic polycistronic translation model, which occurs predominantly in prokaryotes, describes the expression of multiple proteins from one mRNA. With the development of high-resolution proteogenomic techniques, the common model of monocistronic eukaryotic translation, where one mRNA consists of one single open reading frame (ORF), is about to change. A growing number of transcripts that encode for more than one protein, e.g., via additional translation through an internal ribosome entry site (IRES) or by translational initiation at non-canonical initiation codons, were detected in eukaryotes [1,2,3].

Non-canonical peptides have been neglected from the proteome for a long time as they are generally smaller and less structured than main proteins and thought to be of low relevance in cellular function [4]. Nevertheless, recent advances in proteogenomic identification strategies have revealed thousands of previously unannotated and functionally uncharacterized peptides significantly expanding the size of the functional proteome [5,6]. However, the annotation of the full human proteome is still incomplete [7]. Some studies hypothesize that up to 75% of the genome can be transcribed and theoretically translated, potentially offering a large pool of previously unexplored peptides [7,8].

This review aims to give an overview of newly identified non-canonical peptides, particularly peptides encoded upstream of annotated proteins from the same transcript (uPeptides). In the first part, we give a brief overview of different non-canonical peptide categories and describe the state-of-the art technologies in proteogenomic peptide characterization. The second part describes some of the so far identified uPeptides and their functional roles in cellular pathways. We picture different malignant mechanisms that may lead to the expression of novel uPeptides and HLA uLigands. Moreover, we highlight their potential role during tumorigenesis and novel options in developing peptide- and HLA uLigand-based therapeutic strategies in cancer.

## 2. Classification of Non-Canonical Peptides and State-of-the Art Proteogenomic Technology 

In recent studies, pervasive translation outside of canonical coding sequences has been demonstrated mapping to a multitude of possible initiation sites, such as long non-coding RNAs (lncRNAs), 5′-TLS, 3′-UTR, and intronic, intergenic, and off-frame regions [9,10,11]. Expression of those peptides represents a large yet mostly unexplored part of the proteome, often called the “dark” proteome [12,13]. As there is currently no consensus for a uniform definition of non-canonical peptides, we list a selection of frequently used classifications in Table 1 [14,15,16,17,18,19].

As most non-canonical peptides are of short length and low abundance with high turnover rates, their identification has been challenging [18,21]. Because AUG-Methionine is not always used for eukaryotic translation [22], the computational prediction of potential translation initiation sites has become quite complicated. Ribosomal initiation at near-cognate translational start sites (CUG, UUG, GUG, AAG, ACG, AGG, AUA, AUC and AUU triplets) may result in the translation of in-frame as well as out-of-frame ORFs relative to the main coding sequence (CDS) of a specific transcript [23,24]. Over the past few years, several studies developed ranking scores to predict the functional importance of non-canonical ORFs based on conservation as well as on other sequence- and expression-related information [25,26,27]. Bioinformatic scoring of potential non-canonical initiation sites is helpful in selecting ectopic ORFs or peptides for experimental research. However, at present, such computational predictions do not supersede individual experimental validation, as actual translational initiation sites may differ from the predicted ones [17]. Comprehensive searchable databases have been constructed by integrating annotated protein sequences and possible non-canonical ORF sequences, such as the ORF finder from NCBI [28], smProt [29], OpenProt [10], and uORFdb [30]. Databases using a combination of computational prediction, ribosome profiling, and mass-spectrometric (MS) data to map non-canonical translational events in different eukaryotic species revealed thousands of previously unrecognized peptides and significantly increased the quality of proteogenomic screens. The following paragraphs give an overview of major transcriptomic and proteogenomic approaches of non-canonical peptide detection (Table 2).

### 2.1. Ribosome Profiling

Since mRNA translation is a major rate-limiting step in protein synthesis and is highly regulated, there was a need to develop a technique that would allow monitoring the proportion of actually translated ORFs. Ingolia et al. presented a ribosome profiling strategy that was based on deep sequencing of ribosome-protected mRNA fragments and enabled high-precision investigation of protein translation at single-codon resolution [31,32]. Over the years, ribosome profiling has become a powerful tool in the detection of translation initiation sites distinct from annotated protein start codons and revealed several N-terminally extended protein isoforms as well as multiple newly identified regions of translational activity [33,34,35,36,37,38]. 

In principle, ribosome profiling is based on the detection of mRNA molecules that are bound to ribosomes and thereby protected from mRNA degradation at a given time. High-throughput next-generation sequencing (NGS) of those ribosome-protected mRNA fragments provides a “snapshot” of actively translated parts of mRNAs. Several further refinements made the technique more easy to handle, reduced false-positive rates [37,39], and enabled investigation of mRNA translation from multiple species and under varying cellular conditions [40]. Specific pre-treatment strategies prior to ribosome profiling helped to discriminate translational initiation events from ribosomal elongation (GTI-seq) [5,35]. By using the translational inhibitors cycloheximide (CHX) and lactimidomycin (LTM) in combination with ribosome profiling, Lee et al. identified 16,863 potential start sites out of about 10,000 transcripts from human embryonic kidney (HEK293) cells [5]. While CHX inhibits both initiating and elongating ribosomes, LTM only binds initiating ribosomes and makes it possible to differentiate initiating from elongating ribosomes. Another treatment combination consisting of puromycin to inhibit elongating ribosomes and LTM was used to generate a transcriptome-wide map of translation initiation sites (TISs), suggesting 2994 novel ORFs in the 5′ TLS, including 1406 overlapping with the coding sequence (CDS), and 546 N-terminal protein extensions in leukemic THP-1 cells [41]. 

Improvements in analysis of ribosome profiling data increased non-canonical peptide detection. As shown for the translation of canonical CDS, the nucleotide diversity increases periodically every three nucleotides until a roughly equal proportion of each nucleotide is reached [42]. In order to improve the identification of non-canonical ORFs, this periodicity was used to predict novel translating ORFs extending the annotated proteome with approx. 5000 novel ORFs in both wheat and cotton genomes [43].

Although ribosome profiling gives a comprehensive picture of translational activity at ORFs, the method is limited in monitoring the complete proteome, as not every translational event necessarily produces a functional peptide/protein. Therefore, experimental validation of actually expressed peptides is indispensable to provide evidence for their potential relevance in cellular and tumor biology.

### 2.2. Mass Spectrometry-Based Identification of Polypeptides

Mass spectrometry (MS) is probably the most powerful and sensitive proteomic method for non-canonical ORF discovery and has emerged as a standard technique to directly detect the encoded polypeptides [44]. The basis for peptide identification from mass spectrometry spectra is a well-constructed database used to compare the experimentally detected mass-spectra with in silico-predicted digestion and fragmentation libraries of peptide/protein sequences. Large-scale proteogenomic studies made efforts to generate sample-specific databases for MS by focusing on specific regions, including mRNA UTRs [45] or sequences that are predicted to be actively translated based on a combination of ribosome profiling and RNA sequencing data [21]. In proteomics, the liquid chromatography–mass spectrometry (LC–MS) is most frequently used [46]. Proteins from lysed tissues or cell lysates are fractionated and processed by trypsin-mediated enzymatic digestion into peptides. Subsequently, the resulting peptide mixture is positively charged (ionized) and separated according to their mass-to-charge ratio. In a tandem MS approach, the peptides undergo multiple rounds of fragmentation, separation, and detection, resulting in specific spectra [47]. Recent developments in LC-MS workflow, such as improvements in peptide enrichment techniques, have allowed for the identification of 762 non-canonical ORFs from lncRNAs in human and mouse tissues [9]. The combination of different MS-based strategies, including de novo sequencing strategies, led to the discovery of 1074 micropeptides from murine liver, brain, spleen, kidney, and heart [48].

In spite of overwhelming evidence for their translation, the detectability of non-canonical translation products by standard MS-based proteomics using tryptic digestion has been limited [6,49,50], even if specifically adopted isolation methods and peptide libraries have been applied [12,51]. Non-canonical peptides were considered to be of low abundance and to undergo fast proteasomal fragmentation [18]. However, large fractions of those peptide fragments are non-covalently bound by major histocompatibility complexes (MHCs), preventing them from further degradation. By comparing cryptic peptides and canonical proteins, non-canonical peptides showed a lower stability, a comparable translation efficiency and, somewhat surprisingly, a 5-fold higher efficiency of MHC-I processing per translation event [21]. Immunogenic cell surface markers including the MHC-bound peptides and the intracellular proteome can be separated prior to protein lysis [52,53]. Due to reduced background noise of low abundant peptide fragments, MS-based analysis of the MHC-bound peptidome appears to be more efficient for the identification of non-canonical peptides as compared to whole-cell proteomics since it allows capturing peptides with a short half-life time as part of the immunopeptidome [6,20,54,55]. 

The initial experimental determination of the non-canonical translatome (RiboSeq) and the immunopeptidome in patient-derived melanoma cells led to the identification of 456 non-canonical peptides [56]. Other data revealed widespread translation and presentation of cryptic peptides representing approximately 15% of detected human MHC epitopes within the human leukocyte antigen (HLA) system [6,18,21]. In addition, Ruiz Cuevas et al. combined the RiboSeq-based translatome with the MS-based immunopeptidome and the whole-cell proteome, leading to the identification of 2503 new non-canonical peptides in diffuse large B-cell lymphoma [21]. Another remarkable MS-based analysis of the HLA-I immunopeptidome of 29 human primary and cancer cell lines revealed 3555 novel non-canonical ORFs [49]. A comprehensive screening approach named the HLA Atlas project identified 233,053 ligands from 227 benign human tissue samples including 1407 HLA ligands from non-canonical genomic regions [57]. This dataset allows for an accurate comparison of benign and malignant human immunopeptidomes and may help to identify tumor-associated HLA ligands, which are of great interest for the development of new immunotherapeutic strategies in cancer therapy [18]. 

**Table 2:** Recent advances in peptide detection methods.

Technique		Benefit
**Ribosome profiling**	Global translation initiation sequencing (GTI-seq) [5]	Treatment with lactimidomycin or harringtonine prior to ribosome profiling leads to improved detection of ribosomal initiation sites.
	Quantitative translation initiation sequencing (QTI-seq) [41]	Combined treatment with lactimidomycin and puromycin prior to ribosome profiling allows to distinguish between elongating and initiating ribosomes.
	Poly-ribo-seq [37]	Isolation of polysomes; determination of the sequence bound by each ribosome reduces the number of false-positives.
	Translation complex profiling (TCP-seq) [38]	Detection of complete translation cycles; captures differences in translation initiation in carcinogenesis.
	Ribosome nascent-chain complex-bound RNA sequencing (RNC-seq) [36]	Ribosome profiling of mRNAs bound to the ribosomal complex enables analysis of ORFs that are translated at the moment.
**Mass spectrometry**	Liquid chromatography [46]	Liquid chromatography is used to separate mixtures with multiple components mostly followed by mass spectrometry providing spectral information that may help to identify each separated component or confirm the suspected identity of them.
	MHC-based MS [56]	MHC complexes non-covalently bind peptide ligands, protecting them from degradation; enhances the detection sensitivity of non-canonical peptides.
**Experimental validation**	Endogenous peptide tagging [58]	Genomic tagging of peptides using CRISPR/Cas9-mediated HDR visualizes natural expression pattern.
	Split protein tags [59,60]	Self-complementing proteins can be split between the 10th and 11th ß-helix and fused to the peptide, reducing potential side effects of larger tags.
	Co-immunoprecipitation (co-IP) [6]	Detection of peptide–protein interaction by immunoprecipitation of the tagged peptide and bound interactors.
	Pooled CRISPR screen [6,61]	High-throughput screening for functional peptides based on detectable changes in relevant signaling pathways upon CRISPR/Cas9-mediated peptide knockout.
	Perturb-Seq [62,63]	Combination of CRISPR/Cas9-mediated peptide knockout with single-cell RNA sequencing detects changes in RNA-sequencing profiles caused by specific peptide losses.

### 2.3. Individual Detection and Functional Characterization of Non-Canonical Peptides

Multiple CRISPR/Cas9-based techniques were applied to systematically discover non-canonical peptide function in diverse cellular pathways. The application of pooled CRISRR knockout screens using custom single guide RNA (sgRNA) libraries allowed the depletion of thousands of non-canonical peptides. By detecting changes in cellular phenotypes with respect to proliferation, differentiation, apoptosis, or migration upon peptide knockout, several functional peptides could be identified [4]. In a recent large-scale screening approach, CRISPR/Cas9 experiments revealed 57 peptides that induced viability defects when knocked out in human cancer cell lines [61]. The combination of CRISPR screenings and single-cell RNA sequencing (Perturb-seq) allowed for the identification of changes in RNA-sequencing profiles across multiple biological pathways [62,63] and uncovered non-canonical peptides that take part in transcriptional regulation [6,61]. Of note, the induced knockouts in CRISPR/Cas9 screens are partly unspecific, as variable parts of the uPeptide sequences may be deleted or variably repaired by the inclusion of random nucleotides. Consequently, it is difficult to distinguish if additional regulatory motifs, structures, or ORFs are deleted that may have contributed to the observed functional impact, indicating a limitation of this method. A targeted genomic knockout or the introduction of a translation ablating mutation at non-canonical ORF start sites would allow us to specifically modify peptide expression, yet individual approaches are complex and time-consuming processes. The application of a homologous repair template that carries the specific variant during CRISPR/Cas9 approaches could be used to induce the homology-directed repair (HDR) mechanism, leading to integration of the desired mutation at specific genomic/transcriptomic positions [64,65]. 

Antibody-based detection is a powerful tool to map and functionally examine non-canonical peptides, because it allows us to perform experiments in physiological cellular contexts and at the endogenous protein expression level. In the case of non-canonical peptides, due to their short length and low number of structural motifs, the design of a specific antibody may often be difficult and time consuming [66]. Another way to detect non-canonical peptides within the cell is epitope tagging by adding a C- or N-terminal tag to the peptide of interest. Exogenous expression of a tagged peptide expression vector can be used for peptide identification in immunoblot, fluorescence microscopy, and co-immunoprecipitation (co-IP) assays in a variety of cell types. Ectopic V5 tagging revealed evidence for the expression of 257 non-canonical peptides in HEK293T cells [61]. Due to RNA expression analysis, 401 novel peptides inducing changes in gene expression patterns upon overexpression in melanoma, breast, renal, and lung cancer cells were identified [61]. A more reliable way to determine whether a non-canonical peptide is actually translated in vivo is to insert the epitope tag into the genomic locus of the peptide via CRISPR/Cas9-mediated HDR [58]. Sometimes, peptide tagging may be beneficial by increasing protein solubility and proper folding [66], but often, the use of large peptide tags can be problematic, because epitope tags of equal or greater sizes than the peptide of interest may potentially disturb the natural peptide folding, localization, and interaction with other proteins [67]. To minimize those side effects, small protein tags can be applied, including self-complementing split protein tags such as split fluorescent tags [59] or split SNAP tags [60], which have become important labeling tools in protein detection. 

## 3. Functional Implications of uORF-Encoded uPeptides and HLA uLigands

Non-canonical peptides can be encoded by a multitude of possible initiation sites across the genome. A major fraction of non-canonical translation occurs at upstream open reading frames (uORFs) potentially encoding for so-called uPeptides [18,41,45]. Due to the high prevalence of uORF-associated translational activity and strong evidence for frequent uPeptide translation, we focus on the mechanistic and functional implications of uORF-encoded uPeptides for the remainder of this review. 

According to sequence analyses, non-canonical uORFs, initiated by upstream AUG codons or by near-cognate alternative translational initiation sites (aTIS), can be observed in virtually all 5′-transcript leader sequences (TLSs) across eukaryotic species [5,25,41,68,69]. Briefly, translation of a uORF may result in both translational regulation of the associated downstream CDS and/or expression of a uPeptide with potential regulatory functions in *cis* and *trans*. While in general, the presence of AUG uORFs has been associated with reduced CDS expression, the translational regulatory function of an individual uORF on CDS translation is much less predictable. The uORF-mediated functional impact depends on a complex interplay of transcript-specific features, including the length, number, position, and the RNA/peptide sequence as well as the sequence context surrounding the uORF initiation and termination codons [24,35,70]. Main protein expression in uORF-bearing transcripts requires leaky scanning across the uORF start site(s) or reinitiation of ribosomes after translating the uORF followed by reloading with essential co-factors [71,72]. Upstream ORFs play critical roles in diverse cellular programs including the integrated stress response (ISR) [71,73], circadian timekeeping [74,75], and microtubule organization [76]. Translational regulation allows for immediate responses to changing environmental conditions, bypassing the need for time-consuming transcription of new mRNAs. Accumulating evidence of uORF-associated genetic variability suggested an important role of uORF-mediated translational control in several human diseases [24,70,77,78,79,80,81] and during viral infections [82,83]. 

**Figure 1:**
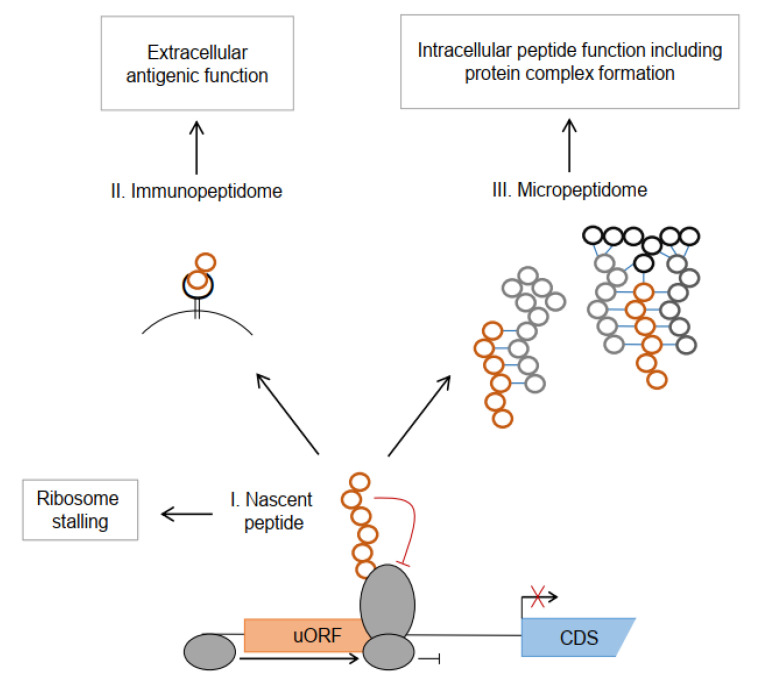
Intra- and extracellular functions of human uORF-encoded peptides. The nascent peptide (**I**) can induce ribosome stalling, mostly followed by transcript degradation, resulting in reduced translation of the main protein CDS, indicated by the crossed out arrow. After proteasomal degradation and processing via the MHC-related antigen presenting machinery, uPeptides contribute to the immunopeptidome (**II**). The uPeptides contribute to the micropeptidome (**III**) and may affect diverse cellular functions through interaction with key regulatory proteins or as part of protein complexes.

The uORF-encoded uPeptides act at the transition of translational regulation and individual uPeptide function (Figure 1). Several uPeptides are described to regulate downstream translation and transcript stability through nascent peptide-induced ribosome stalling across multiple species [84,85,86]. Others are stably expressed and released to the cytosol, contributing to the micropeptidome of cells and acting as individual regulatory peptides or within larger protein complexes. Ultimately, uPeptides undergo proteasomal degradation and are processed by antigen-presenting machinery and exposed at the cell surface within HLA complexes as HLA uLigands. As the functional characterization of individual uPeptides is a laborious task, it has been performed only for a minor fraction of them. In the following paragraphs, we describe examples of regulatory and functional uPeptides with a specific emphasis on their known or anticipated implication in carcinogenesis.

### 3.1. Translational Regulation and Transcript Stability

**Figure 2:**
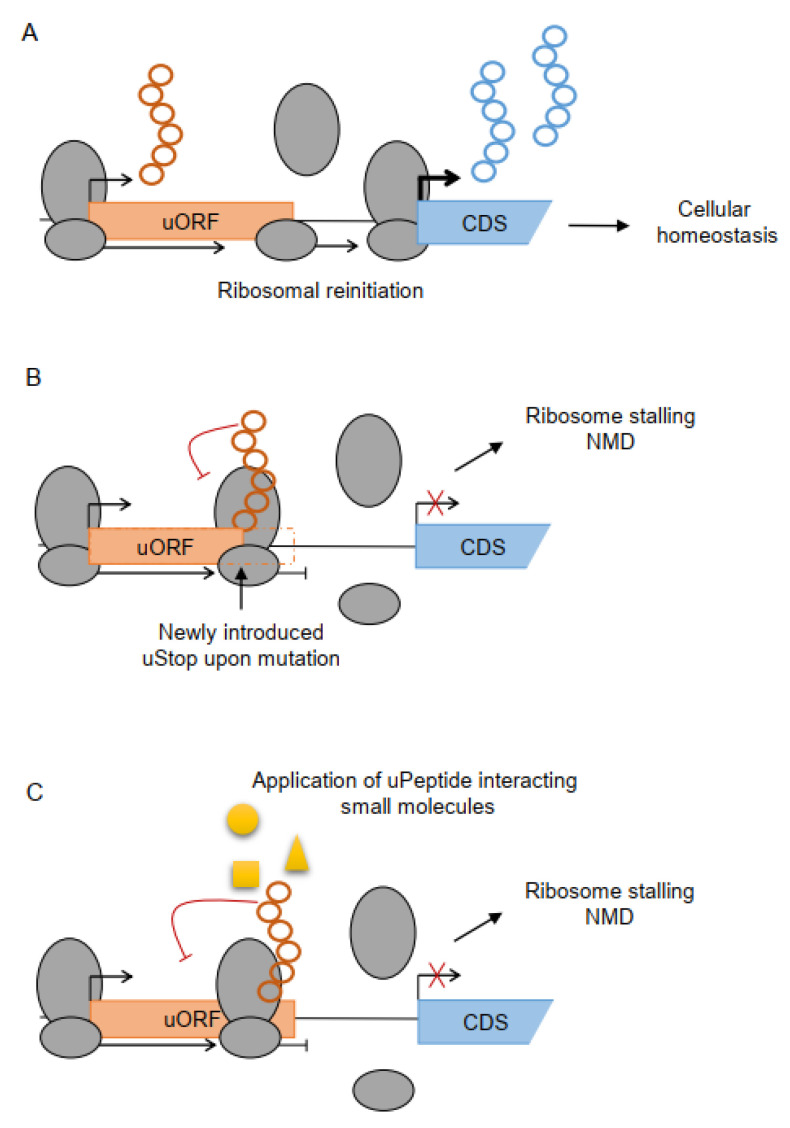
Mechanisms of ribosome stalling-mediated translational regulation. (**A**) Under physiological conditions, ribosomes may scan through the uORF start codon (leaky scanning) or reinitiate at the CDS after translating the uORF, maintaining normal CDS translation and cellular homeostasis. (**B**) Interaction of the nascent peptide with small molecules, metabolites or other molecular interactors may induce ribosome stalling, preventing ribosomal reinitiation, and leading to transcript degradation via nonsense-mediated decay (NMD). (**C**) Mutation-associated introduction of a new uStop codon within the uORF sequence may lead to ribosome stalling and can prohibit downstream translation, indicated by the crossed out arrow.

According to the widely accepted model of cap-dependent translation, the ribosome scans down the mRNA starting from the 5′-cap-structure until it recognizes a suitable initiation site to start translation and protein expression (Figure 2A). While accumulation of aberrant proteins has been associated with a wide range of disturbed cellular functions and several diseases [87,88,89], diverse mechanisms of quality control [90] have evolved to protect cells from uncontrolled protein production or accumulation. Quality control often relies on the eukaryotic translation machinery and may take place even in advance of ribosomal translation during mRNA capping, polyadenylation, and splicing [91,92,93]. During elongation of the nascent peptide, ribosomal pausing is one of the most efficient control mechanisms and may occur upon inhibitory mRNA secondary structures [94], stretches of rare or difficult-to-decode codons [95], mRNA truncation [96,97], and poly(A) sequences [98,99,100]. Frequently, ribosomal pausing occurs during uORF elongation or ribosomes are arrested at uStop codons [101], as recently shown on tryptophan codons upon oxidative stress [102]. Prolonged pausing of ribosomal elongation can result in ribosomal stalling, where subsequent ribosomes queue up behind the pausing ribosome. Besides nascent protein degradation and ribosome recycling [96,103,104,105,106,107], the stalled ribosomes also trigger the nonsense-mediated mRNA decay (NMD) pathway, leading to degradation of the entire mRNA in most cases [97,108,109,110]. Even ribosomal re-initiation at the CDS start after uORF termination does not necessarily protect the mRNAs from NMD [111]. Determination of reporter mRNA half-life time and mining available mRNA stability datasets [112,113] revealed that neither uORF length nor re-initiation efficiency, but rather pausing translation is the main cause of TLS-stimulated mRNA decay [111].

Since error-free ribosomal translation is important for cellular homeostasis, malfunctions of the translational quality control mechanisms disturb cellular homeostasis and have been identified in the pathogenesis of several diseases, including cancer. Recently, Lee et al. showed that variants introducing new stop codons in uORFs (uStops) are under strong negative selection and reduce CDS expression, probably caused by ribosome stalling (Figure 2B) [114]. A previously published example of a variant in the LENG8 TLS that inhibited translation elongation resulted in reduced translation events of the mRNA, supporting the assumption that the occurrence of upstream termination codons and the subsequent premature termination of translation in uORFs may also activate NMD [115]. The association of variants disrupting uORF translation elongation or strengthening uStop codons with human disease in general [114] implies that such uORF-related variants may reduce the expression of tumor suppressor genes, potentially resulting in tumor formation or progression. These ideas call for a reanalysis of cancer sequencing data to search for variants affecting uORF elongation and termination and to evaluate their individual functional impact. 

Several uPeptides are known to induce ribosome stalling upon specific metabolite concentration within the cell (Figure 2C). Small molecules can interact with the nascent peptides and cause stalling of the ribosome, prohibiting further elongation and main protein expression, as recently exemplified for a new class of uORFs that act in response to intracellular levels of copper [116]. Similar examples have previously been reviewed [85], and a selection of metabolite/small molecule-sensing uPeptides is summarized in Table 3. The use of uPeptide interacting molecules may open up a new treatment strategy in human cancer. Potentially, specific metabolites, small molecular or peptide drugs may be able to induce ribosome stalling and NMD at the nascent uPeptide chain selectively upstream of proto-oncogenes. Future studies may systematically search for such uPeptide interacting cofactors able to specifically induce ribosome stalling and ablate translation of harmful downstream oncogenic proteins.

### 3.2. Novel uPeptides May Serve as Immunogenic Antigens 

**Figure 3:**
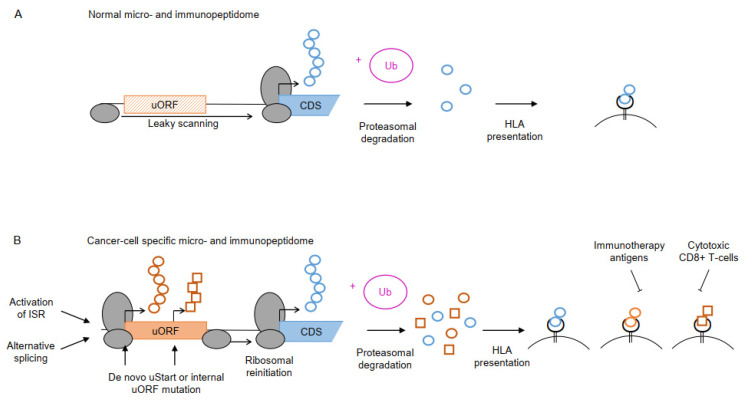
Non-canonical uPeptide-derived neoantigens. (**A**) A large fraction of cellular proteins undergo proteasomal degradation upon ubiquitylation (+Ub) and will partly be presented on the cell surface as HLA ligands. (**B**) Upon tumorigenesis, diverse mechanisms including the activation of the ISR or alternative splicing may enable translation of previously skipped or non-existing uORFs (orange box). Somatic mutations in de novo uORF start (uStart) sites or internal uORF sequence may result in novel uPeptides or changes in uPeptide sequences, altering the micropeptidome compared to healthy cells. Proteasomal degradation of the novel uPeptides generates cancer cell-specific HLA complexes, which may be targetable by immunotherapeutic antigens or cytotoxic T-cells, indicated by inhibitory arrows.

After proteasomal degradation of intracellular proteins or peptides, the HLA class I and class II complexes [128,129] present the processed peptide fragments on the cell surface. Due to enormous genomic variability, the HLA complexes can bind a broad range of peptides and play a pivotal role in the adaptive branch of the immune system (Figure 3A). Abnormal cellular peptides derived from viral infection or malignant transformation encode for neoantigens that are displayed via the HLA-I complexes recognizable for cytotoxic CD8+ T cells and Natural Killer (NK) cells inducing immune responses (Figure 3B). Similarly, alteration of essential cellular pathways such as proliferation control, apoptosis, invasion, and metastasis, altered stress response, and transcriptional re-programming upon malignant transformation change the composition of the immunopeptidome. Such tumor-specific changes may lead to altered uORF translation and uPeptide expression, resulting in differential proteasomal processing and a cancer cell-specific presentation of uPeptide-derived HLA ligands (HLA uLigands) [130,131,132].

The cancer-associated ISR induced by microenvironmental stress drives the translation of specific mRNAs supporting survival, migration, and apoptosis [133]. Xiao et al. suggested a conserved mechanism of deregulated uORF translation in cancers, as exemplified for the ATF4 gene in non-small-cell lung cancer (NSCLC) [134]. There, translation of ATF4 was shown to be remarkably enhanced in NSCLC due to a reduced number of ribosomes binding to the ATF4 uORFs, functionally promoting enhanced cell growth. Another study demonstrated translational upregulation of ATF4 expression in HER2-positive breast cancer cells, resulting in increased cell migration [135]. The protein kinase eukaryotic initiation factor 2 alpha (eiF2a) plays an important role in translation initiation at aTIS codons, which is limited in normal cells. By activation during the ISR, the protein level of eiF2a is frequently upregulated in cancer, especially in squamous cell carcinomas, leading to increased translation of aTIS-uORFs in oncogenic mRNAs [136] (Figure 3B). Due to the altered translation of uORFs in cancer cells, the cancer-related immunopeptidome of HLA uLigands may also change. Another source of non-canonical uPeptide expression and HLA uLigand presentation is differential pre-mRNA splicing [137], which is commonly disturbed during tumorigenesis [138,139,140]. Importantly, alternative splicing not only affects the main protein coding region of a transcript but also the TLS and 3′ UTR, potentially giving rise to new uORFs, deleting preexisting uORFs, or altering initiation, termination, and Kozak sequences. Therefore, uPeptide-derived neoantigens may originate from alternative pre-mRNA splicing in response to malignant transformation. The resulting changes in uPeptide expression and the associated alteration of the immunopeptidome may allow us to discriminate transformed from healthy cells, as recently observed [17]. Additionally, cancer-associated somatic variants lead to the generation of novel uPeptides that may serve as neoantigens (Figure 3B), similar to neoantigens that arise from altered main proteins [141,142]. Recent observations of high somatic variability of uORF sequences suggest a yet largely unexplored contribution of non-canonical ORF-associated genetic variants in shaping the immunopeptidome and immunogenicity of malignant tissues [78,143]. 

Recently, it was sought to identify such cancer-derived neoantigens by comparing the immunopeptidomes from patient-derived malignant and benign tissues, leading to the identification of 31 HLA uLigands exclusively or predominantly detected on malignant cells [17]. As this analysis included only a limited number of approximately 2000 uORF sequences, the large abundance of more than 2.4 million AUG- and aTIS-initiated uORFs [30] in the human transcriptome implies that future studies may uncover numerous additional tumor-specific HLA uLigands. 

In conclusion, there are various mechanisms resulting in a tumor-associated or sometimes even tumor-specific non-canonical micro- and immunopeptidome in cancer. These neoantigens represent highly promising candidates as novel biomarkers and for the development of immunotherapy-based treatment approaches [144]. Several of such HLA-presented neoantigens have already been shown to induce T-cell responses [145,146]. The ability of cytotoxic T cells to specifically recognize and eliminate tumor cells based on specific HLA-I-bound peptides may be utilized for the development of cancer-specific immunologic treatment approaches by vaccination or adoptive T-cell strategies.

### 3.3. Individual Modes of uPeptide Function 

To date, only a minor fraction of uPeptides detected as HLA ligands in immunopeptidomic datasets have been functionally analyzed. Nevertheless, from a limited number of cases, a broad range of individual modes of uPeptide function has been documented (Table 3). Labeling of uPeptides with fluorescent protein tags revealed specific subcellular uPeptide localization varying from ubiquitous distribution of the ASDURF/ASNSD1 uAUG.3 uPeptide, to membrane-associated localization of the MKKS uAUG uPeptide, or the formation of nuclear foci observed for the MAPK1 uCUG.1 uPeptide [17,124]. The number of functional uPeptides is steadily increasing, and we describe several well-characterized examples in more detail below.

As uPeptides often include two or fewer secondary motifs [147], it was suggested that they frequently exert their regulatory function via interaction with larger proteins. This is exemplified by the uPeptides encoded from the HAUS6 and the MIEF1 transcripts, respectively [6,122]. Functional analysis of the HAUS6 uPeptide revealed interaction together with the canonical HAUS6 protein in the HAUS protein complex. Confocal microscopy revealed that the uPeptide localizes at the centrosomes comparable with other HAUS6 complex members [148]. Consistently, the overexpression of HAUS6 uPeptide led to efficient pull-down of other HAUS complex proteins, and a CRISPR/Cas9-mediated knockout arrested the cells at G1 stage. Thus, the uPeptide was shown to be part of the HAUS complex and to be involved in microtubule attachment to the kinetochore and in central spindle formation [6]. Similarly, a uPeptide encoded by the AUG.3 uORF of MIEF1 localizes to the mitochondria, consistent with the localization of the MIEF1 main protein, which regulates mitochondrial fission and fusion [6]. A knockout of MIEF1 uPeptide showed induced expression of mitochondrial fusion and fission genes and led to a tubular and more elongated mitochondrial phenotype (increased fusion). In contrast, its overexpression induced a fragmented mitochondrial phenotype (increased fission). As confirmed by absolute quantification, the MIEF1 uPeptide was found to encode for the predominant protein message instead of the canonical CDS from their shared mRNA [122], assuming how important the uPeptide function can be in cellular biology. 

Multiple functions were assigned to the uPeptide encoded by AUG.3 from the TLS of ASNSD1 (ASDURF), which is ubiquitously expressed in HEK293T cells. Deletion of the uORF led to enhanced main protein expression detected by in vitro dual luciferase assays [17]. As described by Cloutier et al., the uPeptide is involved in a large chaperone complex essential for the assembly and stabilization of other macromolecular complexes, the so-called PAQosome [118]. As a 12th subunit, ASDURF assembles with previously described subunits forming the prefoldin-like chaperone complex [118], which is involved in the assembly and maturation of multi-protein complexes in mammalian cells [149]. In an immunopeptidome screen, the uPeptide was presented predominantly on MHC complexes isolated from leukemia samples, assuming a potential role as a cancer-specific HLA uLigand [17] (Figure 3B). 

**Figure 4:**
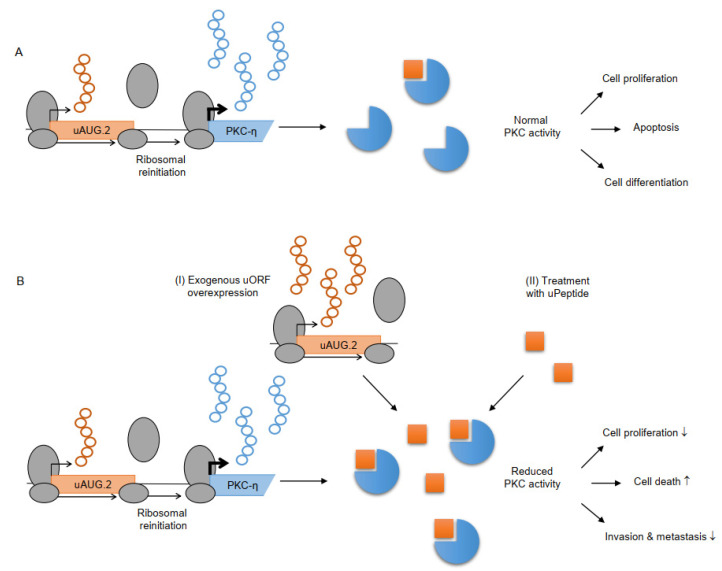
Effects of PKC-η uAUG.2 on protein kinase activity and downstream function [125]. (**A**) The uAUG.2 peptide (orange square) encoded from the PKC-η TLS inhibits the catalytic activity of novel PKCs (blue circles). (**B**) Increased uPeptide levels can lead to reduced PKC activity, resulting in decreased (↓) cell proliferation, invasion and metastasis and increased (↑) cell death. Overexpression of uAUG.2 (I) or direct application of the AUG.2 uPeptide as a small peptide drug (II) may offer novel ways of protein kinase-inhibition in cancer therapy.

Recently, a potential cancer-inhibitory function of the uPeptide encoded by the AUG.2 uORF of protein kinase C-eta (PKC-η) was proposed [125]. PKC-η is a unique member of the protein kinase family and plays critical roles in cell proliferation, differentiation, and cell death [150,151]. The PKC-η AUG.2 uPeptide directly binds and selectively inhibits the catalytic activity of novel PKCs, but not that of classical or atypical PKCs (Figure 4A). In different breast cancer models, overexpression of the uPeptide was shown to suppresses tumor progression, proliferation, invasion, and metastasis and enhance cell death [125]. Exposure of cells to uAUG.2 diminished cell survival and synergized with chemotherapy by interfering with the DNA damage response. The exogenous expression of the uAUG.2 inhibitory uPeptide or the direct application of the AUG.2 uPeptide as a small drug may represent new options for therapeutic protein kinase inhibition in cancer (Figure 4B).

Conclusively, several uPeptides (Table 3) show critical functions involved in transcription [6], translation [121], the JAK-Stat pathway [49], or correct protein folding [118]. Moreover, uPeptides are described to maintain mitochondrial homeostasis [6,124] and inhibit or interact with the downstream encoded main protein [6,125]. Although the abovementioned cellular pathways are frequently implicated in tumorigenesis, a direct oncogenic function of uPeptides has not yet been described. However, novel peptides encoded by non-canonical initiation sites have been found to be specifically expressed in cancer and to show tumor-promoting activities [6,18,21,152]. Those non-canonical peptides act in tumor-associated pathways, promoting proliferation of breast cancer cells [61], supporting translational initiation at selective oncogenes [153], or forming tumor-associated splicing variants in the nucleus [154]. Considering that functional analyses have been performed for only a few uPeptides, we assume that these examples justify intense future work and individual experimental characterization on the large number of functionally unexplored uPeptides to better understand their contribution to cellular homeostasis and to malignant transformation.

## 4. Conclusions and Outlook

The observation of widespread non-canonical peptide expression from regulatory sequences of mRNAs challenges the classical view of eukaryotic transcripts as being mostly monocistronic. Such cryptic peptides, and especially uORF-derived uPeptides, are increasingly recognized to affect multiple cellular pathways, and they constitute a relevant part of the HLA-presented immunopeptidome in humans. The notion of biologically active uPeptides also extends the functional implication and biological relevance of uORFs that have predominantly been considered as translational regulators of downstream main protein expression. The examples described in this review show that translational regulation based on uORFs and encoded nascent uPeptides appear to be highly relevant for cellular homeostasis. Disturbed uORF translation upon reduced availability of ribosomal co-factors or as a consequence of acquired mutations may contribute to human disease and may promote malignant transformation [131,135]. We speculate that in analogy to several uPeptides able to stall ribosomes in response to specific metabolites, future comprehensive drug screening approaches may identify specific small molecule or peptide inhibitors that interact with nascent uPeptides to induce ribosome stalling upstream of oncogenic proteins. 

A multitude of cell biological changes are induced upon malignant transformation, including somatic mutations, changes induced by the ISR, and differential mRNA splicing. Thereby, novel peptides are released to the cytoplasm, processed by the MHC machinery and presented at the cell surface as part of the immunopeptidome. These neoantigens may represent diagnostic biomarkers [144], and several lines of evidence indicate that HLA uLigands may also serve as promising immunotherapy targets [6,17], similar to classical neoantigens, as recently described for the KRAS G12D mutation in pancreatic cancer [141]. Cytotoxic T-cells could be reprogrammed for chimeric antigen receptor T-cell (CAR-T cell) therapy to recognize such cancer-cell-specific uLigands and to ablate the malignant cell clone [155]. 

The biological function of the vast majority of uPeptides currently remains obscure, and their role in tumor biology is not sufficiently understood. However, elaborate individual experimental analyses have identified uPeptides with regulatory functions comparable to other non-canonical peptides or canonical proteins, highlighting the need for a comprehensive characterization of uPeptide function in larger scales. CRISPR/Cas9-mediated knockout screens have begun to pinpoint several candidate uPeptides awaiting deeper functional testing [61,62,63], while other occasional examples such as the uAUG.2 peptide from PKC-η [125] illustrate how small uPeptides may be applied in tumor treatment. Small peptide drugs have been applied for various cancer types [156,157], showing high target selectivity and minimal immunogenicity at the same time [158]. However, the bioavailability and stability of those small peptide molecules will have to be addressed to ultimately facilitate the therapeutic use of uPeptides as small-molecule inhibitors [125,158]. 

In conclusion, uPeptides and HLA uLigands have emerged as a novel class of functional peptides in both healthy and malignantly transformed cells. A better understanding of their cellular function is of interest for the development of new therapeutic approaches via direct targeting or by exploiting their immunogenic capacity for vaccination or CAR-T cell-based immunotherapy.

## Figures and Tables

**Table 1:** Summary of the heterogeneous nomenclature and definition of non-canonical peptides.

Classification	Definition	Site of Initiation
**short peptides** [14]	Peptide-chain with a length of 2–45 amino acids	smORFs, altORFs, uORFs, dORFs, lncRNAs, circRNAs
**small proteins; SEPs** [15,16]	Proteins of less than 100 amino acids in eukaryotes	smORFs, sORFs, altORFs, uORFs, dORFs, lncRNAs, circRNAs
**uPeptides** [17]	Peptides encoded by ORFs in the TLS of main proteins	upstream ORFs
**3′ UTR peptides** [20]	Peptide encoded by ORFs from the 3′ UTR	downstream ORFs
**cryptic peptides** [18]	MHC presented epitopes from non-coding regions	5′ TLS, 3′ UTR, non-coding RNAs, intronic, intergenic and off-frame regions
**miPeps** [19]	Peptides encoded by micro RNAs	miRNAs, pri-miRNAs

smORFs—small ORFs; sORFs—sORFs; altORFs—alternative ORFs; uORFs—upstream ORFs; dORFs—downstream ORFs; lncRNAs—long non-coding RNAs; circRNAs—circular RNAs; SEPs—sORF-encoded peptides; miRNAs—micro RNAs; pri-miRNAs—primary micro RNAs.

**Table 3:** Examples of functional uPeptides in humans.

uPeptide Designation	Genomic Position in hg38	Size	Class	Known Function	Sample Origin
**ADRB2** [117]	chr5: 148826730– 148826790	19 aa	individual uPeptide function	The uPeptide acts as an inhibitor of the hormone receptor Beta-2 adrenergic receptor.	COS-7 cells
**ARAF** [49]	chrX: 47561218– 47561248	9 aa	HLA uLigand	The uPeptide is directly presented by MHC-I complex, does not need protease degradation or processing.	B721.221, A375, HCT116 cells CLL, GBM, Mel samples
**ARL5A** [6]	chr2: 151828232– 151828403	56 aa	HLA uLigand	Detected as an HLA uLigand, shows distinct localization from main CDS.	HEK293T, iPSCs, K562 cells
**ASDURF** [17,118]	chr2: 189663925– 189666058	40 aa	tumor-enriched HLA uLigand; part of protein complex	Takes part in the PAQosome, a chaperone complex. HLA uLigands encoded from ASDURF are predominantly detected on leukemic cells.	HEK293T cells AML, CLL samples
**ASS1** [119]	n.d.	n.d.	individual uPeptide function	The uPeptide regulates expression of ASS1 in a trans-suppressive manner.	BAEC cells
**ATF5 uAUG.2** [17]	chr19: 49929395– 49930906	59 aa	tumor-associated HLA uLigand	Detected as tumor-associated HLA uLigand.	HEK293T cells CLL samples
**CPA1** [120]	n.d.	24 aa	nascent peptide	The uPeptide is responsible for translational repression of CPA1 in presence of arginine.	Yeast strains
**DDIT3** [6]	chr12: 57517711–57520480	34 aa	HLA uLigand; part of protein complex	Detected as an HLA uLigand, forms stable complexes with the main protein involved in transcriptional regulation.	HEK293T, iPSCs, K562 cells
**EPHX1 uORF1** [121]	chr1: 225810536–225828726	26 aa	individual uPeptide function	Expression of EPHX1 is inhibited by trans-acting uPeptides through interactions with the translation machinery.	HEK293A, HEK293T, HepG2, HepG2-C3A, A549 cells
**EPHX1 uORF2** [121]	chr1: 225810576–225828739	17 aa	individual uPeptide function	Expression of EPHX1 is inhibited by trans-acting uPeptides through interactions with the translation machinery.	HEK293A, HEK293T, HepG2, HepG2-C3A, A549 cells
**FBXO9** [6]	chr6: 53065478– 53065673	64aa	HLA uLigand; part of protein complex	Detected as an HLA uLigand, forms stable complexes with the main protein involved in ubiquitination and proteasomal degradation.	HEK293T, iPSCs, K562 cells
**HAUS6** [6]	chr9: 19102714–19102762	15 aa	HLA uLigand; individual uPeptide function	The uPeptide is part of the HAUS6 complex and is involved in microtubule formation.	HEK293T, iPSCs, K562 cells
**HMGA2** [6]	chr12: 65824916–65825123	68 aa	HLA uLigand	Forms a complex with the downstream canonical protein encoded on a shared mRNA.	HEK293T, iPSCs, K562 cells
**LUZP1 uORF1** [49]	chr1: 23094263–23094296	10 aa	HLA uLigand	Ubiquitous expression of the HLA uLigand in 29 primary healthy and cancer samples and cell lines.	B721.221, A375, HCT116 cells CLL, GBM, Mel samples
**LUZP1 uORF2** [49]	chr1: 23094325– 23094376	16 aa	HLA uLigand	Tissue-specific expression in multiple samples of chronic lymphocytic leukemia.	B721.221, A375, HCT116 cells CLL, GBM, Mel samples
**MAPK1 uCUG.1** [17]	chr22: 21807834–21867642	110 aa	tumor-associated HLA uLigand	HLA uLigands encoded by the uPeptide were identified on primary malignant samples and show specific intranuclear localization.	HEK293T cells Mel samples
**MIEF1** [122,123]	chr22: 39504230–39504443	70 aa	part of protein complex	The uPeptide localizes in mitochondria, involved in transcription of mitochondrial fission and fusion proteins.	HEK293T, iPSCs, K562 cells
**MKKS** [124]	chr10: 10420545–10420737	63 aa	individual uPeptide function	The uPeptide localizes to the mitochondrial membrane and is predicted to function independently of the main protein.	HeLa, HepG2, U2-OS, HT1080 cells
**PKC-η uAUG.2** [125]	chr14: 61321953–61322034	26 aa	individual uPeptide function	The uPeptide binds and inhibits the catalytic activity of novel PKCs; overexpression was shown to reduce cancer cell growth and survival.	MCF-7, MCF-10A, MDA-MB-231, U251 MG cells
**SAMDC** [126,127]	chr6: 110874795–110874816	6 aa	nascent peptide	Expression of the CDS is regulated by polyamines binding to the nascent uPeptide; orthologous to A. thaliana SAMDC.	CHO cells
**SOCS1 iORF** [49]	n.d.	n.d.	HLA uLigand	The internal out-of-frame uORF (iORF) encoded peptide is expressed from the SOCS1 gene, a key modulator of interferon and JAK-STAT signaling.	B721.221, A375, HCT116 cells CLL, GBM, Mel samples
**SOCS1 ouORF** [49]	n.d.	n.d.	HLA uLigand	The overlapping uORF (ouORF) encoded peptide is expressed from the SOCS1 gene, a key modulator of interferon and JAK-STAT signaling.	B721.221, A375, HCT116 cells CLL, GBM, Mel samples
**TBPL1** [6]	chr6: 133953303–133980088	42 aa	HLA uLigand	Detected as an HLA uLigand, shows distinct cellular localization from the main protein.	HEK293T, iPSCs, K562 cells
**TMEM203 uAUG.1** [17]	chr9: 137205244–137205640	131 aa	tumor-associated HLA uLigand	HLA uLigands encoded by the uPeptide were detected. The uPeptide shows individual cellular localization.	HEK293T cells AML, CLL, MEL, OvCa samples

n.d.—not defined, meaning that mapping of the published uPeptide sequence with the current hg38 genome assembly failed; ALL—acute lymphocytic leukemia; CLL—chronic lymphocytic leukemia; GBM—glioblastoma, Mel—melanoma; OvCa—ovarian carcinoma.
